# The WEPP Model Application in a Small Watershed in the Loess Plateau

**DOI:** 10.1371/journal.pone.0148445

**Published:** 2016-03-10

**Authors:** Fengpeng Han, Lulu Ren, Xingchang Zhang, Zhanbin Li

**Affiliations:** 1 State Key Laboratory of Soil Erosion and Dry-land Farming on the Loess Plateau, Institute of Soil and Water Conservation, Northwest A & F University, Yangling, Shaanxi province, China; 2 Institute of Water Resources and Hydro-electric Engineering, Xi’an University of Technology, Xi’an, Shaanxi province, China; Beijing Normal University, CHINA

## Abstract

In the Loess Plateau, soil erosion has not only caused serious ecological and environmental problems but has also impacted downstream areas. Therefore, a model is needed to guide the comprehensive control of soil erosion. In this study, we introduced the WEPP model to simulate soil erosion both at the slope and watershed scales. Our analyses showed that: the simulated values at the slope scale were very close to the measured. However, both the runoff and soil erosion simulated values at the watershed scale were higher than the measured. At the slope scale, under different coverage, the simulated erosion was slightly higher than the measured. When the coverage is 40%, the simulated results of both runoff and erosion are the best. At the watershed scale, the actual annual runoff of the Liudaogou watershed is 83m^3^; sediment content is 0.097 t/m^3^, annual erosion sediment 8.057t and erosion intensity 0.288 t ha^-1^ yr^-1^. Both the simulated values of soil erosion and runoff are higher than the measured, especially the runoff. But the simulated erosion trend is relatively accurate after the farmland is returned to grassland. We concluded that the WEPP model can be used to establish a reasonable vegetation restoration model and guide the vegetation restoration of the Loess Plateau.

## Introduction

Soil erosion has become one of the global environmental hazards that limit today's human survival and thus restricts global socioeconomic sustainable development. The global area of soil erosion covers about 16.43 × 10^6^km^2^, or 10.95% of the total area [1]. One of the most serious soil erosion regions in the world is the Loess Plateau, where water erosion impacts more than 45% of the area [2]. Soil erosion has not only caused serious local impacts, but also significant downstream impacts. It has been calculated that the average soil erosion modulus is about 5000–10,000 t km^-2^ and the highest erosion modulus even reaches 20,000–30,000 t km^-2^[3]. Severe soil erosion led to a large amount of sediment discharged into the Yellow river and its tributaries. Approximately 90% of the sediment in the Yellow River originates from soil erosion on the Loess plateau [4]. Some studies have confirmed the reduction of soil erosion on hill slopes or in the small catchments of the Loess Plateau [5, 6]. Fortunately, the recent research showed that the soil erosion on the Loess Plateau decreased significantly and that was caused by the improved vegetation cover and the ecological construction [7]. The implementation of the “Grains for Green Project” which is the largest land retirement project since 1999 has generated substantial environmental benefits and has greatly improved the degraded ecosystems in the Loess Plateau [8]. Much work has been done on soil erosion assessment at plot or catchment scale [9, 10], however, the quantitative assessment of spatially distributed soil erosion has not been adequately addressed, and more work should be done on the soil erosion prediction.

Therefore, it is critical to find an effective soil erosion prediction model and develop a reasonable and scientific soil erosion control program. The WEPP model, the latest generation of soil erosion prediction models, was widely used abroad and involved in many research dfforts [11–14]. However, in China the WEPP model is not commonly used. At present, WEPP’s adaptability has been assessed in the purple soil region of Sichuan Basin [15]. Ye et al.[16] has analyzed the adaptability of WEPP model simulated soil erosion in the soft rock region and concluded that simulation results were better in lands other than fallow land. In the Loess Plateau region, Wang et al. [17] has studied the effect of slope length on the WEEPP model simulation in the gully region. Fang et al. [18], taking Xiang Yang Gou watershed of Yanan area as the research area, concluded that the WEPP model is more reasonable for the small watershed soil erosion simulation, but did not perform well at land use change. Following calibration WEPP Hillslope, slope and watershed versions were compared to observed watershed runoff and sediment delivery observations evaluate the WEPP’s to give an insight of the adaptability of the WEPP model in the hilly gully regions of the Loess Plateau. The objective of this study was to: (1) Calibrating and validating WEPP at the plot scale. (2) Validating GeoWEPP at the watershed scale, and (3) Using GeoWEPP in a case study to examine its use for conservation planning.

## Materials and Methods

### Study Area

**Fig 1:**
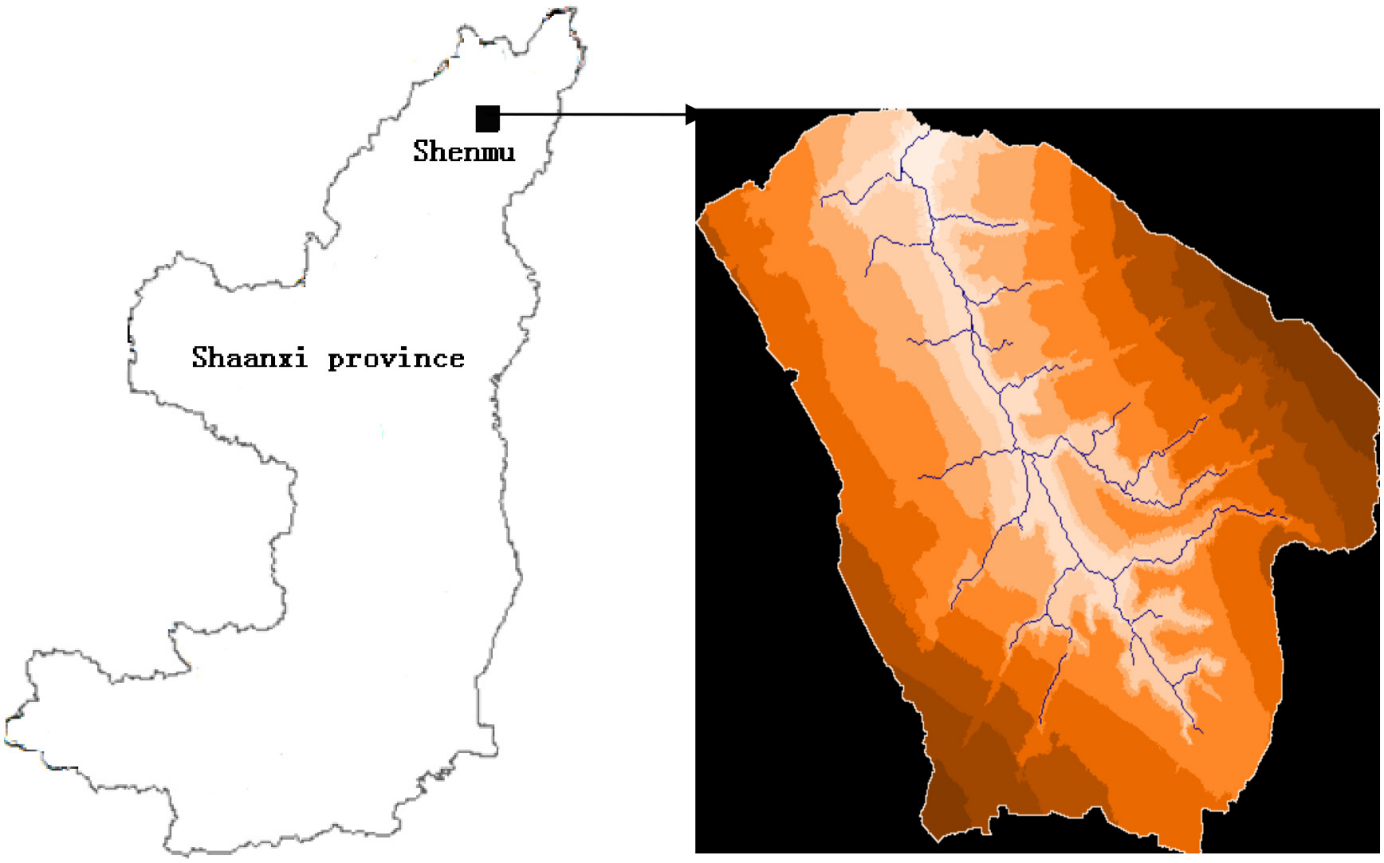
Domain of study area.

The research was conducted in the Shenmu Erosion and Environmental Monitoring Station of Institute of Soil and Water Conservation, Chinese Academy of Science, located in Liudaogou watershed, 14km west of Shenmu (longitude 110° 21'-110° 23 ', latitude 38° 46'-38° 51', and elevation 94–1274 m) (Fig 1). The main north-south channel of Liudaogou watershed is secondary tributary of Kuye River. The watershed has a semi-arid climate, with less rainfall in winter/ spring, and more heavy rain in summer/ autumn. The average annual rainfall is 438 mm (1957–1989) with the maximum rainfall being 892 mm and the minimum being 109 mm. The precipitation is the greatest in July and August, accounting for more than 50 percent of the annual precipitation. The average annual temperature is 8.4°C, the annual accumulated temperature of ≥ 10°C is 3248.0°C, and for 169 days the temperature is ≥ 10°C. The annual average frost-free period is 153 days and the annual sunshine time 2836 h. Northwest winds prevail in late autumn, winter and spring while southeast winds prevail in summer [19]. The study is a 0. 2km^2^ sub watershed within the Liudaogou watershed. In the watershed, eight runoff plots were set up on a loess soil slope. Alfalfa (*Medicago sativa*) was planted and provided coverage amounts of 0%, 20%, 40%, and 60%, two replicates for each coverage. The size of the plots was 1m×2m and the slope of the plots was 0.268 which was the modal slope in the watershed. Two runoff plots were built on a sandy soil slope. The size of the plots was 5m×10 m and the slope of the plots was 0.268. Even the size of the sandy soil plots is bigger than the loess soil plot; there were only two runoff events in the experiment period.

### Model description

The Water Erosion Prediction Project (WEPP) was initiated in 1985, by the USDA [20]. It is a continuous process-based erosion prediction model which can compute runoff and soil loess along a slope. The watershed version of WEPP also can estimate the watershed runoff and sediment yield under different land use and soil types [21]. The WEPP model represents a new soil erosion prediction technology which is based on the fundamentals of infiltration theory, soil physics, hydrology, plant science, hydraulics, and erosion mechanics [20]. It is composed of nine components: weather generation, irrigation, soils, hydrology, plant growth, winter process, residue decomposition, erosion and deposition, hydraulics of overland flow [22]. Like most of the soil erosion model, the erosion component of WEPP is based firmly on a steady state continuity equation which is composed of interrill and rill erosion. Interrill erosion is considered to be independent of distance that is to say interill erosion occurs at a constant rate down the slope. Rill erosion is positive for detachment and negative for deposition. Each of these parameters is calculated on a per rill area basis, thus the sediment load is solved as soil loss per unit rill area. One difference between the WEPP model and other models is that the sediment continuity equation is applied within rills rather than using uniform flow hydraulics [23].

### Meteorological data

The meteorological data with 5 minute interval for 10 years (2000–2010) were collected from the station in the watershed. It was composed of the precipitation, temperature, wind velocity and direction. In the WEPP model, 10 weather daily parameters are required. In this study, the meteorological data from the weather station determined the time to peak intensity, peak 5-min rainfall intensity, precipitation amount and duration, maximum and maximum temperature, wind velocity and direction. The other two parameters (solar radiation and dew point temperature) were generated by the WEPP weather generator, CLIGEN [24].

### Hydrological data

The rainfall data with 5 minute interval were collected from the weather station in the watershed. The annual runoff and soil erosion of the plots and watershed were monitored under natural rainfall to test the adaptability of the WEPP in the loess area. Plastic buckets which could receive the runoff and soil erosion were placed under the outlets of the ten runoff plots. Every rainfall we would change the plastic buckets and measured the runoff volume and sediment concentration. At the same time, sediment yield at the outlet of the watershed were measured. During the experiment time, five runoff events happened in the loess soil plots while two events (Aug 26 and Aug 28) happened in the sandy soil plots (Table A in S1 File). We obtained the runoff volume and sediment concentration of the each events. A weir was installed at the small watershed outlet to monitor the runoff and sediment delivery continuously for a year under natural rainfall conditions to test the adaptability of the WEPP watershed version in the Loess Plateau. In the weir, a float-type water depth gauge (model: XYF3) was equipped. In the ordinary days, there was no runoff. During the experiment period, only two runoff events (Aug 26 and Aug 28) happened. When a runoff event occurred, we started the float-type water depth gauge and collected 500 ml water samples every 5min to estimate the sediment concentration. All these samples were collected by hand and analyzed using filtration, drying and weighting of the dry matter.

### Topographic data

In this study, all of the digital maps were measured by us. First, we circled the different soil types and land use and measured the elevation of the watershed using a Trimble 5700 RTK precision GPS. The elevation of the watershed was measured using continuous method and the distance between two lines is less than 1 m. Then, the soil maps and land use maps were built with the ArcGIS 9.3 software and the digital elevation model (DEM) was obtained with a 1 m resolution for the small watershed (Fig A in S1 File).

### Soil data

The soil samples of these plots were collected with two replicates for each plot. The soil samples of the watershed were from the previous study which collected 169 locations with a 15×15 m grid [25]. The CEC, organic content and soil particle composition of all the samples were analyzed. The soil albedo was calculated using the Baumer equation: soil albedo = 0.6/e(^0.4^*^ORGMAT)^ where ORGMAT is the percentage of organic material content. The Manning’s roughness (n) obtained from the laboratory rainfall experiments (Li and shao, 2008). The saturated hydraulic conductivity (K_s_) was measured with tension infiltrometer and the initial saturation level (%) was 75%. The soil data used in this study are showed in Table B in S1 File. Because there was no plant on the sandy soil, the parameters of the sandy soil were consistent in the plots and watershed.

### Model input parameters

Major inputs for WEPP model include climate data, topography data, soil attributes and management conditions. In the hillslope scale, most of the inputs data of the plots were derived from the runoff plots. The management files derived from the WEPP bare soil management file and the continuous grass file with different coverage. In the watershed scale, because only two runoff events were happened, the parameters remain used from the plot scale calibration and GeoWEPP model was used to subdivide the watershed area into hillslope. The required data in the GeoWEPP are soil layer, land use layer and DEM. The critical source area (CSA) and minimum source channel length (MSCL) that were used to control the size of the sub-catchment area were defined. Finally, 103 hillslope sub-catchments and 41 channels were obtained. These sub-catchment file, land use layer, soil layer and slope information were used in the WEPP watershed model.

### Calibrate and simulate method

One method of ideal model calibration is using data that include a range of conditions [26]. In this study, the calibrations used four plots of loess soil with different coverage and two plots of sandy soil. Then, the validating simulations were performed in the other four plots with different plant coverage, using the calibrated parameters. In these artificial plots, there were five runoff events that were occurred in the experiment period. Every runoff event was simulated by the WEPP model as a single storm. All the calibrated parameters were used in the other four plots simulation and the watershed simulation. The sensitivity analysis of these calibrated parameters has been obtained by decreasing or increasing the values by 10%, 20%, 25% and 50%, respectively and then calculated the sensitivity ratio.

The t test and The Nash-Sutcliffe coefficient were used to verify the accuracy of the simulated results. All these tests were finished by SPSS 16.0 software. The t test significance level 0.05 was postulated: if the p value calculated by t test is greater than 0.05, that means the simulation is accurate. The Nash-Sutcliffe coefficient was determined for comparing the predicted to the observed the runoff and sediment delivery [27]. If NS is negative, it means that the model is not as good at prediction as simply using the mean value of the observed data. If close to zero then, it means the simulated result equals the average. If close to 1, it means that the simulated result is much better than the average. The Nash-Sutcliffe model efficiency (NS) is calculated by:

$$NS=1-\frac{\sum_{i=1}^{n}{\left(Q_{i}-P_{i}{}^{*}\right)}^{2}}{\sum_{i=1}^{n}{\left(Q_{i}-\bar{Q}\right)}^{2}}$$ (1)

## Results and Discussion

### Calibrate and sensitivity analysis

The calibrated parameters of the different soil types in the watershed are presented in the Table 1. The effective hydraulic conductivity (K_e_), interill erodibility (k_i_), rill erodibility (K_r_) and critical shear stress (τ_c_) were obtained from the four loess soil plots and two sandy soil plots. The biggest different parameter between the two soils was K_e_ which was 11.86 mm∙h^-1^ in the sandy soil but 9.06 mm∙h^-1^ in the loess soil. These calibrated parameter values were in agreement with the range of values reported in the WEPP model documentation [28]. A major limitation in this study is that the parameters of the watershed level were the same values calibrated with the measured data at the plots scale due to non-availability of enough measured runoff and sediment data at the watershed.

**Table 1:** The calibrated values and sensitivity ratio of soil parameters based on the WEPP plot runs.

Soil type	wind Sandy soil				Loess soil			
parameter	K_i_ (kg∙s∙m^-4^)	K_r_ (s.m^-1^)	K_e_ (mm∙h^-1^)	τc (Pa)	K_i_ (kg∙s∙m^-4^)	K_r_ (s.m^-1^)	K_e_ (mm∙h^-1^)	τc (Pa)
Value	10412000	0.03659	11.86	3	10412000	0.03397	9.06	3.5
runoff	0	0	-0.104	0	0	0	-0.046	0
Sediment yield	0.002	0.038	-0.172	-0.059	0.003	0.194	-0.033	-0.305

K_e_ effective hydraulic conductivity, K_i_ interill erodibility, K_r_ rill erodibility, τ_c_ critical shear stress.

Sensitivity analysis can be used to judge the degree of the sensitiveness about each parameter. The results of the sensitivity analysis ratio for runoff and sediment yield are presented in Table 1. The results show that the runoff is sensitive to K_e_ alone whereas, sediment yield was sensitive to K_e_, k_i_, K_r_ andτ_c_ as could be seen by the sensitivity ratio values bigger than zero. This result was consistent with the literature [29, 30].

### Validating WEPP at the artificial plot scale

**Fig 2:**
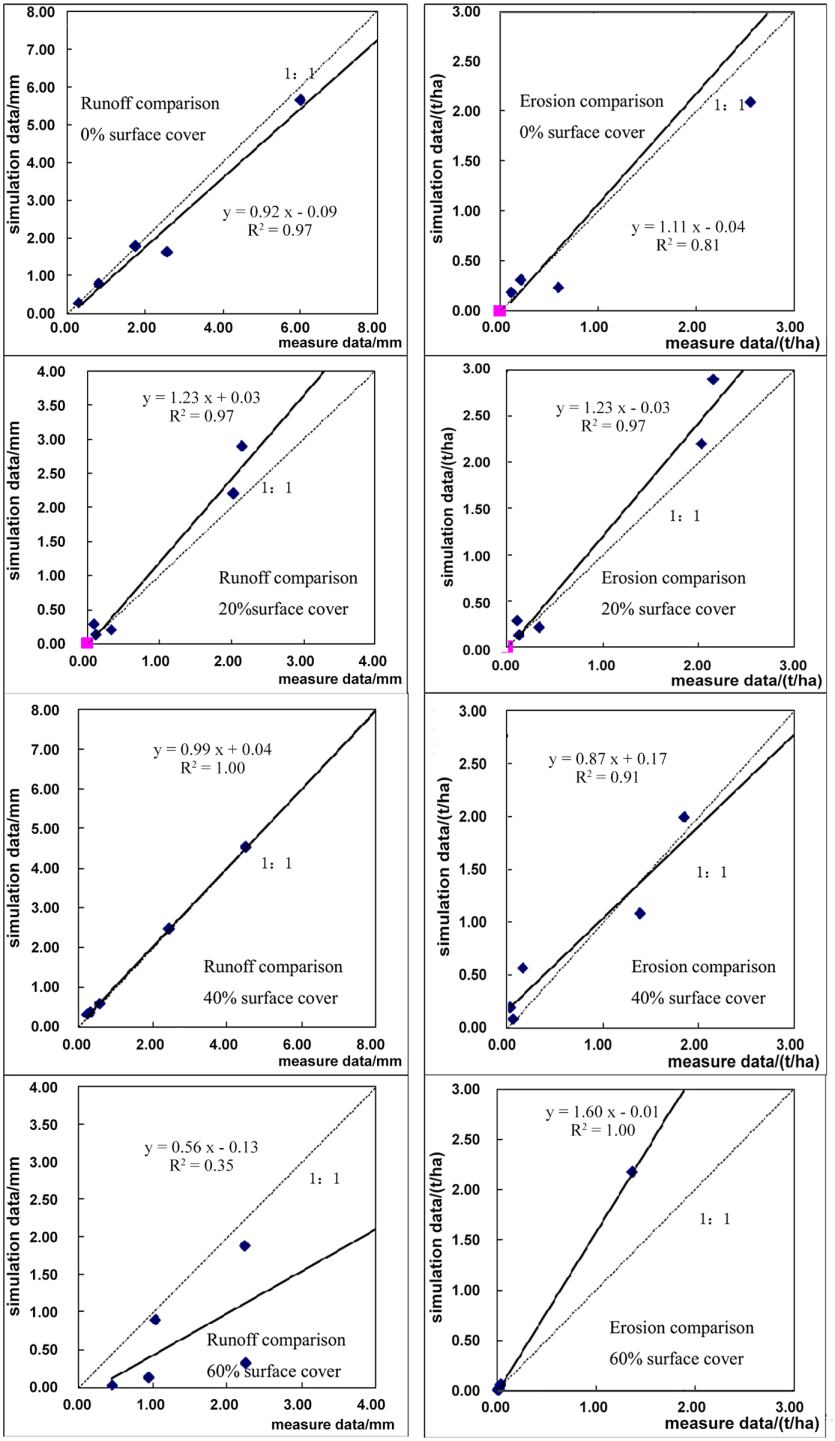
Runoff and Soil Erosion Comparison of Artificial Plots.

The simulated and measured values were compared to understand the adaptability of the WEPP at the artificial plot scale in the Loess Plateau. Fig 2 shows the comparison of the measured and simulated values of rainfall runoff and soil erosion for artificial plots. If the slope of the regression line in the figure is 1, it means that the simulated values equal the measured values. The closer to 1 the slope is, the better the simulated result will be. By comparing the measured and simulated values of runoff and soil erosion under different vegetation cover amounts, the results showed that the WEPP-simulated runoff and sediment yield predictions are relatively consistent with the measured values. Except for the low regression coefficient between the measured and simulated runoff under 60 percent coverage, the regression coefficients between other measured and stimulated values reach 0.8. By comparison with the 1:1 line, it is concluded that the simulated runoff is under predicted for 0 and 60% coverage, and the simulated runoff is over predicted for 20%. For most events, the simulated erosion is slightly higher than the measured. That is caused by the different vegetation coverage. The higher vegetation coverage can reduce the runoff and soil erosion [31]. But the degree of the influence for the runoff is more sensitive than the soil erosion [32]. With the calibrated parameters, when the coverage is 40% which was the common coverage in the watershed, the simulated results of both runoff and erosion are the best. So these parameters are suitable for the watershed simulation.

T-test and Nash-Sutcliffe model were used to verify the accuracy of simulated results. Parameters and results are shown in Table 2. The Nash-Sutcliffe model efficiencies are high for both the runoff and erosion in the artificial plots, which means the simulated results are quite good. Even though the simulated results are not as good under the coverage of 60%, The Nash-Sutcliffe model efficiency is still 0.55, much greater than 0. A two-tailed t-test statistic was used to test the similarity between the observed and predicted runoff and sediment delivery values for each cover amount. The results in Table 2 show that there is less than a 5 percent probability that there is a difference between the predicted and observed values for all cover amounts. This indicates that observed and simulated runoff and sediment are not significantly different at 95% confidence level. The hillslope version of the WEPP model can be used to predict runoff and soil erosion.

**Table 2:** WEPP model performance using erosion plot data.

Test Parameters	Analysis Items	0%	20%	40%	60%
Nash model efficiency	Runoff	0.95	0.97	0.99	0.79
	Sediment	0.70	0.85	0.90	0.55
Paired T-test	Runoff	0.20	0.25	0.10	0.08
	Sediment	0.77	0.273	0.53	0.37

### Validating WEPP at the watershed scale

Through the simulated analysis of the artificial plots using the hillslope version of the WEPP model, various parameters for the model were calibrated and validated. Combined with ArcGIS and GeoWEPP analysis, runoff and soil erosion in small watershed were obtained. A comparison between predicted and observed runoff and sediment delivery values are shown in Table 3, the observed annual runoff from the small watershed is 83m^3^, with a sediment concentration of 97 g/l, this is equivalent to a sediment delivery of 8.057t or an erosion rate of 0.288t / (ha∙yr). Both the simulated values of runoff and erosion were higher than the measured. The predicted runoff was 110 m^3^, 27 m^3^ higher than the observed. This is mainly because that the WEPP model is simulated in the region where the slope is gentle and only slope and rill erosions exist. Zhang et al. [33] carried out theoretical analysis and experimental verification on the rill erosion with steep slope and corrected the parameter calibration. Therefore the simulated results are consistent with the measured for the slope scale is not related to gully unique to Loess Plateau. However not only the slope runoff and sediment yield, but gully runoff and erosion should be taken into consideration when it comes to watershed scale because gully erosion represents an important sediment source and gullies are effective links for transferring runoff and sediment from uplands to valley bottoms [34] Relatively simplistic sediment routing equations in the WEPP model and assumption for static hillslope and channel dimensions during the simulation may be one of the contributing factors for the deviation [22]. The over-prediction of small events and under-prediction of large events is inherent to all erosion models [20, 35]. Another limitation in this study is that the parameters were calibrated with the measured data at the artificial plots due to no enough measured runoff and sediment data at the watershed scale. The hydrology parameters vary with different scales [36]. Table 3 shows that modelers should be cautious when simulating runoff and sediment yield at a small watershed scale to consider the mechanisms and processes of the model, especially the erosion processed of the Loess Plateau gully and should pay more attention to the parameters calibration at different scales in the future.

**Table 3:** WEPP model performance using small catchment data.

Items	Runoff (m^3^)	Erosion (t)	Erosion intensity (t/(ha∙yr))
Measured Value	83	8.057	0.288
Simulated Value	110	10.2	0.35
Deviation	27 (32.5%)	2.143 (26.6%)	0.062 (21.5%)

### Using GeoWEPP to guide the conservation planning

**Fig 3:**
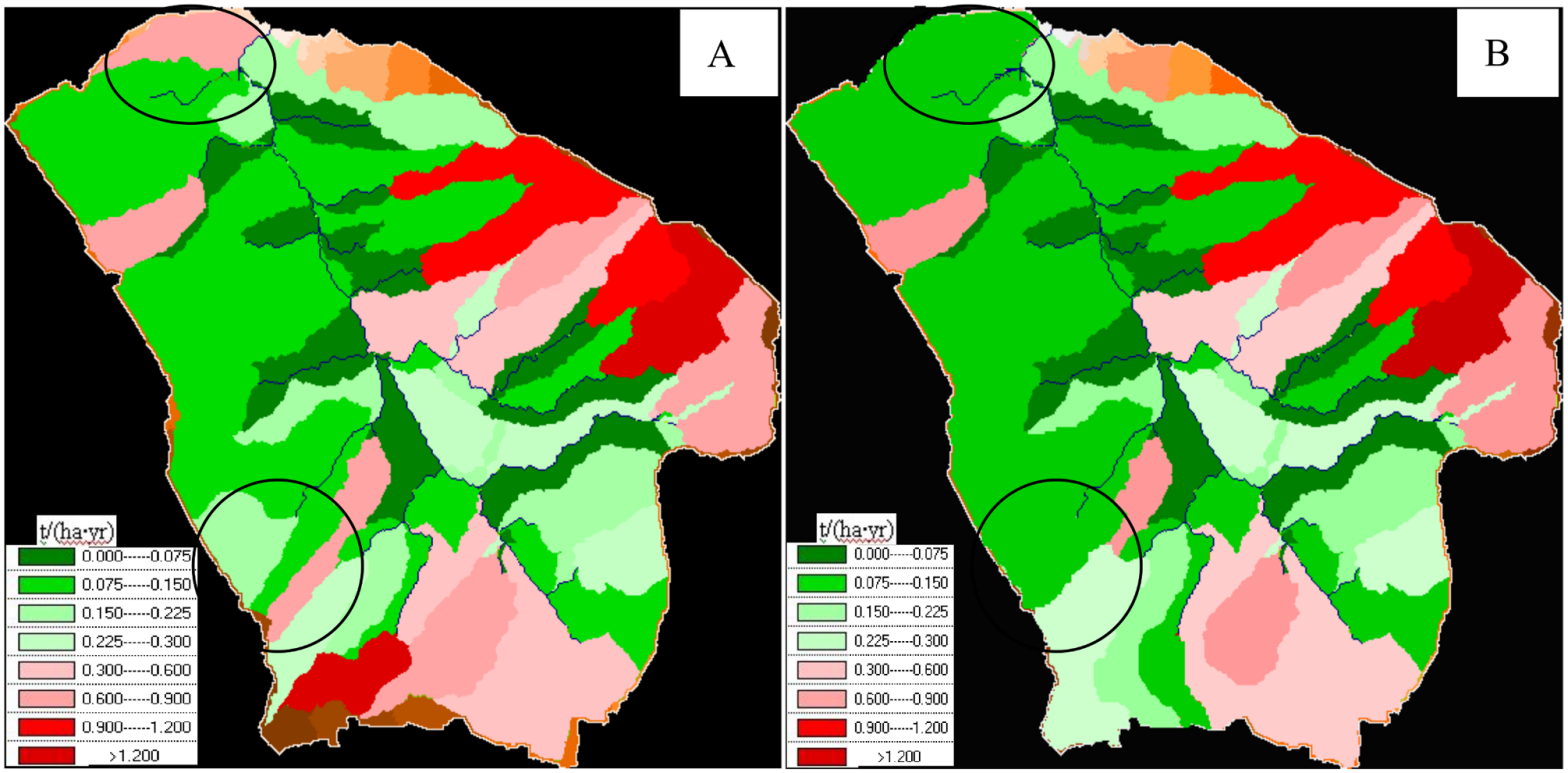
Soil erosion distribution map in small watershed.

With 0.3 t/ha^2^∙yr as the tolerable soil erosion limit, the erosion distribution predicted by GeoWEPP is shown in Fig 3a, where pink and red areas represent the polygons with more than the tolerable sediment delivery. Combined with soil type and land use maps (Fig A in S1 File), it can be seen that the areas with greater erosion are mainly distributed in the areas with relatively high elevation and sparse vegetation. Among different soil types, sandy soil has relatively low erosion because of the hydraulic conductivity (Table 1). The model can simulate erosion distribution due to different soil and land use types, which can be used to plan vegetation establishment, and then reduce erosion through planting vegetation in the areas with the greatest erosion.

A small number of the hillslope in the small watershed are still agricultural lands. Fig 3b shows the GeoWEPP-simulated erosion change before and after returning farmland to alfalfa. Because the agricultural land is small and mainly distributed around the areas circled in black in Fig 3, the changes mainly happen within those areas. If changes to green after the simulated returning farmland to alfalfa, it means it is within the permissible soil erosion and returning farmland to grassland can reduce soil erosion.

The agricultural land of the watershed is 15781m^2^, accounting for 7.89% of the total watershed area. Table 4 shows that the runoff and erosion will drop 2m^3^ and 0.4t respectively and account for 1.82% and 3.92% of the total loss, if the farmland is returned to alfalfa. Because a small proportion of the total area is agricultural land, the change is not great. But the change amount indicates that GeoWEPP model can be used to guide the comprehensive management of the Loess Plateau watershed.

**Table 4:** Comparison between the measured and simulated erosion.

Land management	Runoff (m^3^)	Erosion (t)	Erosion Intensity (t/ha∙yr)
Crops converted to Alfalfa	108	9.8	0.33
Existing land management	110	10.2	0.35

## Conclusions

This study compared observed small plot and small watershed erosion data to erosion predicted by the WEPP model. From this study, the following conclusions can be drawn: (1) in the small plots with flat slope, mainly rill and interrill erosion occurred, and simulated results are similar to the measured; (2)the simulated value at the watershed scale is greater than observed, suggesting some of soil or vegetative properties of the small plots were not typical of the watershed as a whole.; (3) although the WEPP stimulated erosion and runoff values at the watershed scale were greater than observed values, the simulated erosion trends after returning farmland clearly show the benefit of replacing croplands with a perennial forage crop. So it can be used to guide the restoration of Loess Plateau and establish a reasonable vegetation layout mode. Further study on the spatial variability of soil and vegetative cover is needed to successfully model larger areas.

## Supporting Information

S1 File Table A: Characteristics of single event storms. Table B: Soil parameters used for each soil type and treatment in the plots and watershed. Fig A: The land use, elevation and soil GIS layers for the watershed (these map was obtained from our measured GPS data).
(DOCX)
