# Supplementary material for: The WEPP Model Application in a Small Watershed in the Loess Plateau
Source: PLoS One. 2016 Mar 10;11(3):e0148445. doi: 10.1371/journal.pone.0148445 (PMC4786288; doi:10.1371/journal.pone.0148445)
Supplement: S1 File — Table A: Characteristics of single event storms. Table B: Soil parameters used for each soil type and treatment in the plots and watershed. Fig A: The land use, elevation and soil GIS layers for the watershed (these map was obtained from our measured GPS data). (DOCX) [file pone.0148445.s001.docx]

Table A. Characteristics of single event storms

| date | Storm amount(mm) | Storm duration(h) | %Duration to peak intensity |
| --- | --- | --- | --- |
| Aug 3 | 12.3 | 3.22 | 0.68 |
| Aug 26 | 26.4 | 5.17 | 0.45 |
| Aug 28 | 32.9 | 7.25 | 0.36 |
| Sep 01 | 19.7 | 6.03 | 0.51 |
| Sep 13 | 9.2 | 4.92 | 0.79 |

Table B. Soil parameters used for each soil type and treatment in the plots and watershed

| Soil type/treatment | CEC  (meg/100g) | Organic  (%) | Clay  (%) | Silt  (%) | Sand  (%) | albedo | K_s_  (mm﹒min^-1^) | n |
| --- | --- | --- | --- | --- | --- | --- | --- | --- |
| sandy soil | 2.63 | 0.291 | 0.25 | 2.31 | 97.44 | 0.53 | 11.89 | - |
| Loess soil (watershed) | 6.7 | 0.676 | 6.18 | 52.07 | 41.75 | 0.46 | 0.94 | 0.03 |
| Loess soil (Alfalfa 0%） | 6.7 | 0.65 | 5.66 | 51.8 | 42.54 | 0.46 | 0.94 | 0.03 |
| Loess soil (Alfalfa 20%） | 7.2 | 0.76 | 6.56 | 52.84 | 40.60 | 0.44 | 1.25 | 0.058 |
| Loess soil (Alfalfa 40%） | 7.5 | 0.94 | 5.84 | 51.13 | 43.02 | 0.41 | 1.41 | 0.062 |
| Loess soil（Alfalfa 60%） | 8.1 | 1.04 | 6.88 | 51.00 | 42.12 | 0.39 | 1.61 | 0.072 |


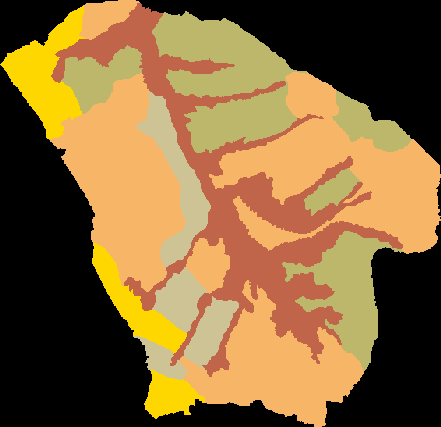

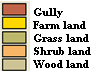

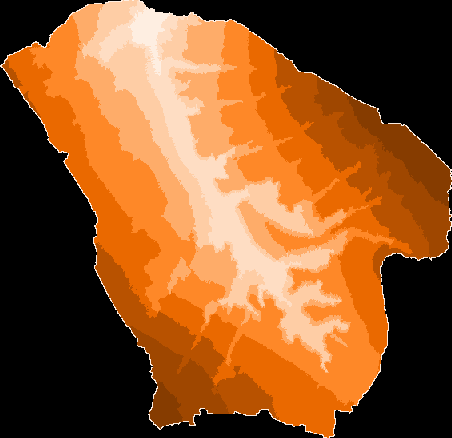

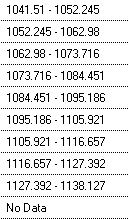

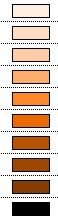


ASCII format of DEM


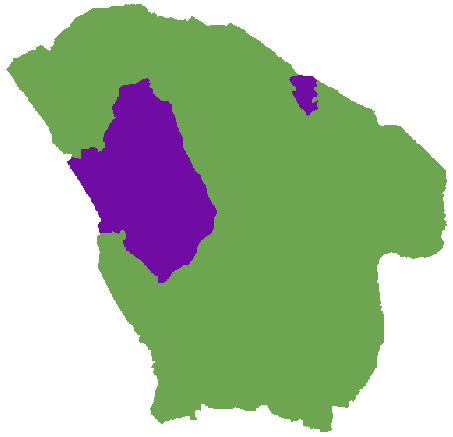

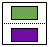

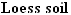

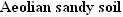


ASCII of soil types

ASCII of land use types

ASCII format of soil types

**Fig A.** The land use, elevation and soil GIS layers for the watershed (these maps were obtained from our measured GPS data using ArcGIS 9.3)
